# Prognostic value of tryptophan catabolism-base scores in acute myocardial infarction patients

**DOI:** 10.1016/j.jare.2025.03.025

**Published:** 2025-03-25

**Authors:** Ye Wang, Pengyan Wu, Zhanchao Chen, Zhaoying Li, Yini Wang, Miao Yan, Yiying Zhang, Shanjie Wang, Shaohong Fang, Bo Yu

**Affiliations:** a State Key Laboratory of Frigid Zone Cardiovascular Diseases (SKLFZCD), China; b Department of Cardiology, Second Affiliated Hospital of Harbin Medical University, China; c The Key Laboratory of Myocardial Ischemia, Chinese Ministry of Education, China; d Department of Epidemiology and Biostatistics, School of Public Health, Jiamusi University, Jiamusi, China

**Keywords:** Tryptophan catabolism, Acute myocardial infarction, Indole-3-propionic acid, Indole-3-lactic acid, Prognosis

## Abstract

**Aims:**

Tryptophan catabolism is implicated in the progression of cardiovascular disease. We sought to investigate the prognostic value of tryptophan catabolism-related features in patients with acute myocardial infarction (AMI).

**Methods and Results:**

A prospective cohort of 4071 patients (mean age: 60.7 years; 69.1 % men) with AMI between February 2017 and June 2019 was included and followed up for a median of 5.6 years (IQR 5.1–6.2). There were 666 all-cause deaths, 365 cardiovascular deaths, and 559 HF events. Plasma levels of tryptophan-related metabolites were measured using liquid chromatography tandem mass spectrometry (LC-MS/MS), and were repeatedly determined in 1044 patients after discharge. Tryptophan, kynurenine, indole-3-propionic acid, and indole-3-lactic acid were screened to construct tryptophan metabolites combination (TMC) score using coefficients from predictive models for MACE. Patients were divided into 3 groups by TMC tertiles. Patients with higher TMC score were older, more likely to be male and have hypertension. Compared to those with TMC tertile 1, patients in TMC tertile 3 had significant associations with the risk of all-cause death (HR: 1.90; 95 %CI: 1.54–2.34), cardiovascular death (HR: 2.32; 95 %CI: 1.71–3.15) and incident HF (HR: 1.77; 95 %CI: 1.40–2.24). The incremental prognostic value of TMC score over the Grace score was measured by the likelihood ratio, C-statistic, continuous net reclassification improvement (NRI), and integrated discrimination improvement (IDI) for prediction, discrimination, and reclassification of outcomes.

**Conclusions:**

In this hospital-based AMI cohort, the TMC score was significantly associated with all-cause mortality, cardiovascular mortality, and incident HF, and improved risk stratification beyond established clinical risk factors. The TMC score provided a novel tool for assessment of Trp catabolism dysfunction and outcomes risk.

## Highlights

• The TMC score was developed to investigate Tryptophan catabolism dysfunction in an AMI cohort.
• The TMC score was significantly associated with all-cause mortality, cardiovascular mortality, and incident HF.
• The TMC score improved risk stratification beyond established clinical risk factors.

## Graphical abstract

(A) Forest plot for association between Trp metabolites and incident MACE. Adjusted for Trp, Kyn, IPA and ILA. 4071 patients with AMI were admitted into the hospital, and 1044 of them went outpatient follow-up. (B) Forest plot for association between Trp metabolites and MACE, adjusted for Trp, Kyn, IPA and ILA. (C) Associations between TMC scores and outcomes. (D) Comparison between two TMC score regression coefficients.

**Figure:**
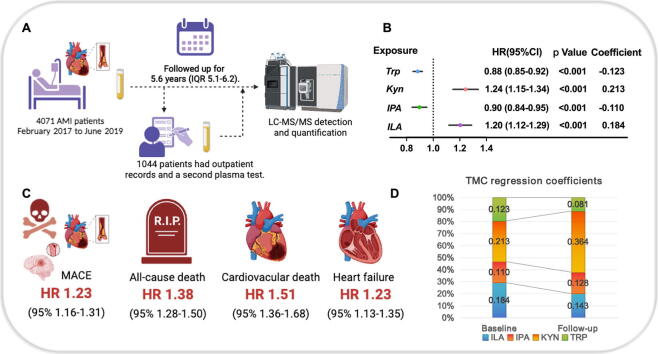

## Introduction

Despite the updating in management, patients with acute myocardial infarction (AMI) are still at high risk for major adverse cardiovascular events (MACE) [1]. Current guidelines recommend that the early risk stratification is essential to guide treatment decisions for patients with AMI [2], [3]. Traditional risk scores including Framingham, Predict equation, and Grace scores are usually composed of clinical variables and biomarkers (cardiac troponin I, B-type natriuretic peptide, and highly sensitive C-reactive protein) [2], [4], [5]. Many studies have reported that dysregulation of the tryptophan (Trp) catabolism was associated with cardiovascular disease (CVD) [5], [6], [7]. Several retrospective studies have suggested associations between Trp level and poor prognosis of AMI [8], [9], [10]. But a few studies found contradictory associations between CVD events and plasma IPA or Kyn metabolites [11], [12], partly due to small sample size and short-term follow-up.

Trp catabolism is mainly divided into three pathways: the kynurenine (Kyn) pathway (95 %), the indole pathway (5 %) and the serotonin pathway (1–2 %) [13]. An accelerated catabolism of Trp in the Kyn pathway was proved to be a marker of cardiometabolic diseases [6], [14]. Serotonergic dysfunction has been considered a myriad of cardiopulmonary pathologies, accelerating adverse remodeling and cardiac dysfunction after AMI [15]. In contrast, higher concentration of metabolite products in indole pathway have shown complicated effects. For example, indole-3-propionic acid (IPA) inhibits atherosclerosis and protects against heart failure (HF) [16], [17], while indole-3-lactic acid (ILA) was associated with increased risk of MACE [18]. However, previous studies mostly focused on single markers at a certain time point, which might not be enough to reflect overall features and the change in the metabolic pathways of Trp.

Given that these individual metabolites exhibited significant biological impact in the progression of CVD, developing a combined score system with multiple biomarkers is crucial. Thus, we quantified seven metabolites in Trp pathway using liquid chromatography tandem mass spectrometry (LC-MS/MS). A scoring system was built based on regression coefficients, referred to Trp metabolites combination (TMC) score. We calculated TMC scores for AMI patients in the cohort and assessed the scores’ 1-, 2- and 3-year MACE risk, mortality and incident HF (Graphical abstract). Moreover, whether adding TMC score as a covariate to the traditional risk models improves the predictive performance was also investigated.

## Method

### Study population

Participants were sourced from a prospective large-scale cohort established by the Second Affiliated Hospital of Harbin Medical University, supported by the National Key R&D Program of China. Detailed protocols have been described elsewhere [19]. Briefly, a total of 5041 patients with AMI were recruited from February 2017 to April 2019, and blood samples were collected on admission and stored in the biobank of the Key Laboratory of Myocardial Ischemia, Ministry of Education. We excluded 130 participants with a history of HF, 30 with thrombolysis therapy, 89 with pulmonary hypertension, 9 with uremia, and 12 with malignant tumors. 314 participants with missing data, 86 participants with probiotic and antibiotic use in 7 days, and 300 participants with no plasma available were also excluded. A total of 4071 patients were included in this study, 1044 of whom came to outpatient follow-up and blood samples were collected for second assessment (Supplemental Fig. 6).

### Ethics statement

All study participants gave written informed consent. The study was carried out in accordance with the Declaration of Helsinki and approved by local Ethics Committees (Approval no. YJSKY2024-118).

### Biomarker measurements

Blood samples were obtained at time of presentation. We analysed plasma samples using LC-MS/MS and quantified seven metabolites: Trp, Kyn, IPA, ILA, indole-3-acetic acid (IAA), serotonin and melatonin. Laboratory personnel blinded to sample allocation and patient data conducted the measurements. Biomarkers analyses and assays used were summarized in the Supplemental Method. We examined recovery, matrix effects, long-term stability, and freeze–thaw stability. Measurement accuracy was in the range of 93.4–107.9 %, inter-day precision was 3.1–13.0 % and intra-assay precision was 2.5–9.3 %.

### Clinical characteristics

Demographic characteristics, lifestyle factors, comorbid conditions, AMI severity and interventions were extracted from medical records. Body mass index (kg/m^2^) was calculated based on weight and height. Smoking status was categorized as either current smokers or nonsmokers. Clinical definitions were applied for diabetes, hypertension, previous myocardial infarction and stroke. Baseline ejection fraction (EF) was determined via echocardiography within 7 days post-AMI attack. The estimated glomerular filtration rate (eGFR) was calculated using the CKD-EPI (Chronic Kidney Disease Epidemiology Collaboration) creatinine equation. The angiographic variables, including culprit vessels and number of diseased vessels, were analyzed. Door-to-ballon (D2B) time was extracted from the medical records.

### Follow-up and study outcomes

The study outcomes were defined as MACE, all-cause death, cardiovascular death, and HF. Participants were followed prospectively until death, or May 31, 2024. Events were identified from inpatient records, periodical outpatient visits and phone call interviews. MACE was defined as all-cause death, cardiovascular death, HF, reinfarction and non-fatal stroke. Cardiovascular death was defined as death from one of the following: myocardial infarction, HF, sudden death, post-resuscitation, vascular cause, during or after cardiac surgery, or during cardiac catheterisation. HF was diagnosed during outpatient or inpatient service, evidenced by symptoms of dyspnea, NT-proBNP levels more than 300 pg/mL, and at least one of the following: treatment with diuretics or intravenous vasodilators, pulmonary rales, lower extremity edema, pulmonary congestion or third heart sounds on imaging, and persistent sinus tachycardia [20]. The diagnosis of all events was further validated through reviewing medical records by two experienced cardiac doctors.

### Statistical analysis

#### TMC score development and evaluation

Spearman correlation coefficients were used to assess correlations between each two Trp metabolites. We used cox proportional-hazards regression models to assess the associations between seven Trp metabolites and MACE, then excluded those were not significantly associated with MACE. We built a scoring system based on regression coefficients, referred to as Trp metabolites combination (TMC) score [21]. All the metabolites were naturally logarithmically transformed to improve discrimination and calibration of the models. To assess the linear association between TMC and outcomes, restricted cubic splines (RCS) models were used. The number of knots was determined based on the Akaike information criterion.

Continuous variables were presented as mean (SD) or median (IQR), and categorical variables as percentages. We used the χ^2^ statistic and student’s *t* test to compare baseline characteristics across TMC score tertiles. Kaplan-Meier curves with log-rank tests assessed the cumulative incidence of post-AMI outcomes according to TMC tertiles. Cox proportional-hazards regression models were used to compute hazard ratios (HRs) and 95 % CIs for the associations between TMC scores and outcomes. Analyses were adjusted for age and sex (Model 1). A fully adjusted model included age, sex, hypertension, diabetes mellitus, smoking status, BMI, previous myocardial infarction, previous stroke, PCI, TnI, NT-proBNP, hs-CRP, and CKD-EPI (Model 2).

Because 1044 patients of survivors after AMI discharge had plasma collected at outpatient visit, we detected plasma metabolites concentrations and reanalyzed their associations with MACE. The regression coefficients were updated as the second TMC score. Then we divided two TMC scores of 1044 patients into high and low groups separately, then combined them into 4 groups. We performed 3 sensitivity analyses to assess the robustness of our findings. First, we excluded patients who died within 30 days and repeated Cox regression analyses. Second, we replaced NT-proBNP with EF for Cox regression analyses. Third, Fine-Gray competing risk models were used to study the association between TMC and cardiovascular death by modeling non-cardiovascular death as a competing risk. All-cause death was considered as a competing risk for HF.

#### Predictive performance analysis

We further evaluated the incremental prognostic value of TMC score beyond the reference model in AMI for outcome risk prediction, which was based on the Grace score and cardiac biomarkers including TnI and NT-proBNP. The GRACE version 2.0 model was used and analyzed as continuous variables [22]. Calibration was assessed via the likelihood ratio test, Akaike information criterion (AIC), and Bayesian information criterion (BIC). Discrimination was evaluated using Harrell's C index and area under the curve (AUC). Reclassification was assessed via continuous net reclassification improvement (NRI) and integrated discrimination improvement (IDI), with 1-, 2- and 3-year cutoffs to determine event status within the Cox model framework. Data analyses were performed using Stata (version 16.0) and R (version 4.3; R Foundation for Statistical Computing, Vienna, Austria). All tests with a two-sided P < 0.05 were considered statistically significant unless otherwise noted.

## Results

### Baseline characteristics and TMC score

A total of 4071 AMI patients were included. The mean (SD) age was 60.7 (11.6) years and 69.10 % of the cohort were male (Table 1). To select potential biomarkers for TMC scores, we examined the plasma levels of seven candidate Trp metabolites. Four of them (Trp, Kyn, ILA, and IPA) were independently associated with MACE after multiple adjustment (Supplemental Fig. 1). Spearman correlation values between these biomarkers were reported in Supplemental Table 1. These findings suggested the feasibility of using them as components for TMC score system. The Cox proportional-hazards regression model coefficients and hazard ratios for MACE events were presented in graphical abstract, with Trp, Kyn, ILA, and IPA adjusted. TMC scores were normally distributed in the total cohort, and relationships between TMC scores and outcomes were linear (Supplemental Fig. 2), then we divided the cohort into TMC score tertiles. TMC scores tended to be higher among males, older participants, smokers, and those with higher hs-CRP levels.

**Table 1:** Baseline characteristics of all AMI patients.

	**Total**	**TMC tertile 1(n = 1357)**	**TMC tertile 2(n = 1357)**	**TMC tertile 3(n = 1357)**	**P Value**
**Demographics**					
Age, years	60.7 ± 11.6	59.3 ± 11.4	60.3 ± 11.4	62.5 ± 11.6	<0.001
Male	2813 (69.10)	917 (67.58)	945 (69.64)	951 (70.08)	0.158

**Cardiovascular risk factors**					
BMI, kg/m^2^	25.0 ± 3.8	24.9 ± 3.7	25.1 ± 3.9	24.9 ± 3.9	0.584
Current smoker	2625 (64.48)	832 (61.31)	878 (64.70)	915 (67.43)	0.001
Diabetes Mellitus	959 (23.56)	325 (23.95)	323 (23.80)	311 (22.92)	0.527
Hypertension	2130 (52.32)	672 (49.52)	699 (51.51)	759 (55.93)	0.001
Total cholesterol, mmol/L	4.57 (3.94, 5.25)	4.57 (3.95, 5.23)	4.57 (3.94, 5.22)	4.57 (3.92, 5.29)	0.953
HDL, mmol/L	1.22 (1.06, 1.42)	1.21 (1.03, 1.39)	1.22 (1.07, 1.44)	1.22 (1.08, 1.43)	0.003
LDL, mmol/L	2.82 (2.28, 3.41)	2.82 (2.32, 3.41)	2.82 (2.26, 3.4)	2.82 (2.28, 3.4)	0.33
hs-CRP, mg/L	5.75 (2.47, 11.71)	5.33 (2.23, 11.27)	5.75 (2.48, 11.55)	6.24 (2.74, 12.4)	<0.001
CKD-EPI, mL/min/1.73 m^2^	81.5 (65.8, 96.9)	89.4 (75.2, 102.4)	82.5 (69.2, 97.1)	71.0 (53.4, 87.3)	<0.001

**Medical history**					
Myocardial infarction	391 (9.60)	121 (8.92)	131 (9.65)	139 (10.24)	0.241
Stroke	877 (21.54)	274 (20.19)	269 (19.82)	334 (24.61)	0.005

**MI characteristics**					
EF, %	57.1 ± 7.8	58.2 ± 6.7	57.3 ± 7.6	55.9 ± 8.9	<0.001
Troponin I, ng/L	33.8 (9.6, 95.9)	29.0 (8.7, 86.6)	33.4 (9.3, 96.0)	37.7 (10.4, 107.4)	0.016
NT-ProBNP, pmol/L	975 (343, 2551)	803 (280, 1951)	900 (344, 2307)	1343 (453, 3930)	<0.001

**Medical treatment**					
Asprin	3955 (97.15)	1332 (98.16)	1333 (98.23)	1290 (95.06)	<0.001
Clopidogrel or ticagrelor	3972 (97.57)	1336 (98.45)	1334 (98.31)	1302 (95.95)	<0.001
Statins	3920 (96.29)	1320 (97.27)	1319 (97.20)	1281 (94.40)	<0.001
β-Blocker	3962 (97.32)	1326 (97.72)	1319 (97.20)	1317 (97.05)	0.285
ACEI/ARB	2976 (73.10)	995 (73.32)	1002 (73.84)	979 (72.14)	0.489
CAG	3928 (96.49)	1309 (96.46)	1319 (97.20)	1300 (95.80)	0.348
PCI	3361 (82.56)	1127 (83.05)	1122 (82.68)	1112 (81.95)	0.448
Follow-up max years	5.6 (5.1, 6.2)	5.6 (5.3, 6.1)	5.6 (5.2, 6.3)	5.6 (4.8, 6.3)	<0.001

**Tryptophan metabolites**					
Trp, μg/mL	9.5 (6.8, 12.7)	8.9 (6.0, 12.0)	9.5 (7.1, 12.5)	10.3 (7.5, 13.5)	<0.001
Kyn, ng/mL	332.8 (249.1, 440.5)	232.5 (185.9, 291.5)	332.8 (280.0, 387.8)	476.3 (387.2, 601.4)	<0.001
IPA, ng/mL	179.0 (88.3, 509.2)	242.5 (103.1, 1149.8)	163.8 (91.2, 419.0)	154.4 (72.1, 360.2)	<0.001
ILA, ng/mL	135.1 (96, 201.8)	93.8 (70.2, 119.7)	135.1 (106.1, 173.5)	225.2 (171.2, 301.6)	<0.001
IAA, ng/mL	12.8 (11.6,15.4)	12.5 (11.3, 15.8)	12.8 (11.6, 15.1)	13.0 (11.9, 15.5)	0.466
Serotonin, ng/mL	9.7 (7.0, 15.6)	9.2 (6.8, 12.7)	9.6 (7.5, 14.8)	10.1 (7.5, 17.1)	<0.001
Melotonin, pg/mL	38.6 (27.7, 54.7)	36.6 (25.3, 56.9)	36.6 (28.8, 49.9)	42.0 (30.1, 56.3)	0.951

**Coronary angiography features**					
**Number of diseased vessels**					
1 vessel, n (%)	1204 (30.65)	395 (30.22)	388 (29.55)	421 (32.19)	0.607
2 vessels, n (%)	1684 (42.87)	569 (43.53)	574 (43.72)	541 (41.36)	
3 vessels, n (%)	1040 (26.48)	343 (26.24)	351 (26.73)	346 (26.45)	

**Culprit vessel, n (%)**					
LAD	1885 (47.99)	589 (45.07)	644 (49.05)	652 (49.85)	0.093
LCX	931 (23.70)	326 (24.94)	315 (23.99)	290 (22.17)	
RCA	1112 (28.31)	392 (29.99)	354 (26.96)	366 (27.98)	
Door-to-balloon (minutes)	69 (50, 87)	69 (50, 88)	69 (49,87)	68 (49, 87)	0.29

Values are mean (SD), median (IQR) or n (%). BMI = body mass index; CKD-EPI = chronic kidney disease epidemiology collaboration; IPA = indole-3-propionic acid; ILA = indole-3-lactic acid; Trp = tryptophan; Kyn = kynurenine; LAD = left anterior descending; LCX = left circumflex; RCA = right coronary artery.

### TMC score and outcomes

**Fig. 1:**
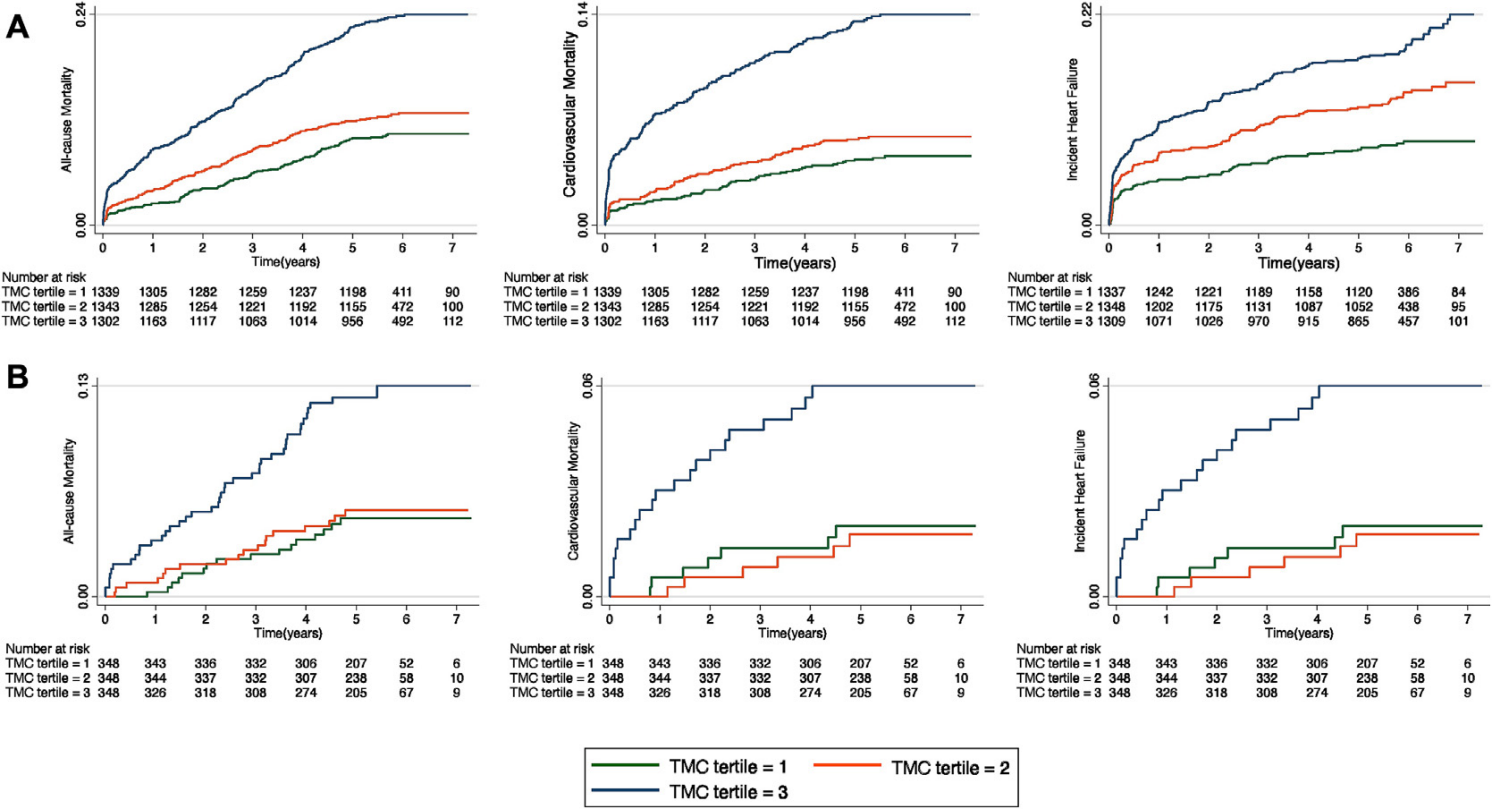
**Kaplan-Meier Survival Curves by TMC score tertiles in AMI patients** (A) Kaplan-Meier survival curves according to TMC score tertiles for all-cause mortality, cardiovascular mortality and incident HF at baseline. (B) Kaplan-Meier survival curves for 1044 patients’ second scores during follow-up according to TMC tertiles for all-cause mortality, cardiovascular mortality and incident HF.

Over a median follow-up of 5.6 years (IQR 5.1–6.2), there were 666 total deaths and 365 cardiovascular deaths. The incidence rates (per 1000 person-years) of all-cause death across TMC tertiles were 18.5 (95 % CI 15.6–21.9), 22.9 (95 %CI 19.6–26.6) and 46.3 (95 %CI 41.4–51.9). Cumulative cardiovascular mortality across TMC tertiles were 8.0 (95 % CI 6.2–10.3), 10.5 (95 % CI 8.4–13.1) and 26.6 (95 % CI 22.9–30.9), respectively. During follow-up period, 559 patients had HF. This corresponded to 15.9 (95 % CI 13.2–19.2), 26.3 (95 % CI 22.7–30.4) and 39.8 (95 % CI 35.0–45.2) HF events per 1000 patient-years. Kaplan-Meier curves showed graded positive associations between TMC score tertiles and all-cause mortality, cardiovascular mortality, and incident HF (each log-rank test < 0.001; Fig. 1A).

Cox regression models were used to examine the associations between TMC scores and adverse outcomes (Table 2). In unadjusted model, TMC scores were positively associated with MACE (HR: 1.38; 95 %CI: 1.31–1.46), all-cause mortality (HR: 1.59; 95 %CI: 1.48–1.71), cardiovascular mortality (HR: 1.77; 95 %CI: 1.61–1.94) and incident HF (HR: 1.44; 95 %CI: 1.33–1.56), with each SD increase. Compared to the lowest TMC tertile, patients in the highest tertile had significantly higher risk for MACE (HR: 2.06; 95 %CI: 1.77–2.38), all-cause death (HR: 2.51; 95 %CI: 2.05–3.07), cardiovascular death (HR: 3.28; 95 %CI: 2.44–4.40) and incident HF (HR: 2.37; 95 %CI: 1.89–2.97). In the multivariable analysis, the associations were slightly attenuated. TMC scores remained associated with MACE (HR: 1.23; 95 %CI: 1.16–1.31), all-cause mortality (HR: 1.38; 95 %CI: 1.28–1.50), cardiovascular mortality (HR: 1.51; 95 %CI: 1.36–1.68) and incident HF (HR: 1.23; 95 %CI: 1.13–1.35), with each SD increase. Patients in TMC tertile 3 had a nearly 2-fold increase in the risk of MACE (HR: 1.63; 95 %CI: 1.40–1.90), all-cause death (HR: 1.90; 95 %CI: 1.54–2.34), cardiovascular death (HR: 2.32; 95 %CI: 1.71–3.15) and incident HF (HR: 1.77; 95 %CI: 1.40–2.24), compared to tertile 1.

**Table 2:** Association between TMC tertiles and outcomes in AMI patients.

	**Tertile 1(n = 1357)**	**Tertile 2(n = 1357)**	**Tertile 3(n = 1357)**	**Ptrend**	**Increase per SD**	**P Value**
**MACE (n = 1212)**						
Unadjusted	1	1.37 (1.17–1.60)	2.06 (1.77–2.38)	<0.001	1.38 (1.31–1.46)	<0.001
Model 1	1	1.33 (1.14–1.55)	1.88 (1.62–2.18)	<0.001	1.33 (1.26–1.41)	<0.001
Model 2	1	1.27 (1.08–1.48)	1.63 (1.40–1.90)	<0.001	1.23 (1.16–1.31)	<0.001

**Death (n = 666)**						
Unadjusted	1	1.25 (0.99–1.56)	2.51 (2.05–3.07)	<0.001	1.59 (1.48–1.71)	<0.001
Model 1	1	1.18 (0.94–1.48)	2.17 (1.77–2.66)	<0.001	1.51 (1.40–1.63)	<0.001
Model 2	1	1.15 (0.91–1.44)	1.90 (1.54–2.34)	<0.001	1.38 (1.28–1.50)	<0.001

**Cardiovascular death (n = 365)**						
Unadjusted	1	1.31 (0.93–1.84)	3.28 (2.44–4.40)	<0.001	1.77 (1.61–1.94)	<0.001
Model 1	1	1.25(0.89–1.76)	2.88 (2.14–3.87)	<0.001	1.71 (1.55–1.88)	<0.001
Model 2	1	1.18 (0.84–1.66)	2.32 (1.71–3.15)	<0.001	1.51 (1.36–1.68)	<0.001

**HF (n = 559)**						
Unadjusted	1	1.62 (1.28–2.05)	2.37 (1.89–2.97)	<0.001	1.44 (1.33–1.56)	<0.001
Model 1	1	1.57 (1.24–1.99)	2.16 (1.72–2.71)	<0.001	1.39 (1.28–1.51)	<0.001
Model 2	1	1.46 (1.15–1.85)	1.77 (1.40–2.24)	<0.001	1.23 (1.13–1.35)	<0.001

The values are hazard ratios (95 % confidence intervals). Model 1: Adjusted for age and sex. Model 2: Adjusted for variables in Model 1 plus hypertension, diabetes mellitus, smoking status, BMI, previous myocardial infarction, previous stroke, PCI, TnI, NTpro-BNP, hs-CRP and CKD-EPI. TMC = Trp metabolites combination.

In the cohort, 1044 patients had two plasma measurements, of which Trp and IPA concentrations showed no difference, but Kyn and ILA levels decreased (Supplemental Fig. 3). The second TMC scores were associated with incident MACE in both unadjusted model (HR: 1.36; 95 %CI: 1.20–1.53) and multi-adjusted model (HR: 1.21; 95 %CI: 1.06–1.38), with each SD increase (Supplemental Table 2). Kaplan-Meier curves showed strong associations between TMC scores and outcomes (each log-rank test < 0.001; Fig. 1B). Then we divided two TMC scores of 1044 patients into high-low stratifications and combined them into 4 groups. In unadjusted model, Cox regression analyses showed that patients with First_high_Second_high_ scores had 78 % higher risk for MACE (HR: 1.78; 95 %CI: 1.33–2.39). After multi-adjustment, patients in First_high_Second_high_ group remained associated with MACE (HR: 1.52; 95 %CI: 1.12–2.07) (Table 3). Consistent outcomes were obtained when we excluded patients who died within 30 days (Supplemental Table 3). Similar outcomes were obtained when we replaced NT-proBNP with EF (Supplemental Table 4). Fine-Gray competing risk regression models showed that the outcomes remained significant (Supplemental Table 5).

**Table 3:** Association between two measurements’ TMC scores high-low groups and outcomes in AMI patients.

	**First_low_Second_low_** **(n = 401)**	**First_high_Second_low_** **(n = 121)**	**First_low_Second_high_** **(n = 121)**	**First_high_Second_high_** **(n = 401)**
**MACE (n = 245)**				
Unadjusted	1	1.34 (0.85–2.11)	1.60 (1.05–2.46)	1.78 (1.33–2.39)
Model 1	1	1.43 (0.91–2.26)	1.54 (1.00–2.36)	1.68 (1.25–2.25)
Model 2	1	1.13 (0.69–1.84)	1.48 (0.96–2.28)	1.52 (1.12–2.07)

The values are hazard ratios (95 % confidence intervals). Model 1: Adjusted for age and sex. Model 2: Adjusted for variables in Model 1 plus hypertension, diabetes mellitus, smoking status, BMI, previous myocardial infarction, previous stroke, PCI, TnI, NTpro-BNP, hs-CRP and CKD-EPI. TMC = Trp metabolites combination.

### The improvements of TMC scores based on the reference model

Finally, we evaluated the incremental value of adding TMC scores as a covariate to reference models including the Grace score, TnI, and NT-proBNP at 1, 2, and 3 years (Table 4). Likelihood ratio (LR) tests showed that the overall goodness of fit improved when TMC scores were added to the reference model (each p ≤ 0.0251). For all outcomes, the Brier scores including AIC and BIC decreased after TMC scores included. We found higher Harrell's C index and higher AUC values, which showed the incremental prediction of TMC scores (Supplemental Fig. 4). TMC scores improved the reference model’s 1-year continuous NRI for MACE (NRI: 0.127 (0.019–0.151)), for all-cause death (NRI: 0.132 (0.015–0.169)), as well as the NRI for cardiovascular death (NRI: 0.308 (0.035–0.410)) and HF events (NRI: 0.174 (0.016–0.235)). The improvements were equally significant at the 2- and 3-year outcomes. As for the relative IDI, it was 0.014 in predicting MACE, 0.010 in all-cause mortality, 0.028 in cardiovascular mortality, and 0.018 in HF at 1 year. The similar results were observed for the 2- and 3-year outcomes. Further, the calibration curves showed high accuracy of the combined model for predicting outcomes (Supplemental Fig. 5).

**Table 4:** Incremental predictive value of adding TMC scores as a covariate to reference models in AMI patients.

**1-year**	**MACE**		**All-cause death**		**Cardiovascular death**		**Heart failure**	
Model	Reference*	Reference + TMC	Reference*	Reference + TMC	Reference*	Reference + TMC	Reference*	Reference + TMC
Likelihood ratio test	−	0.0002†	−	<0.0001†	−	<0.0001†	−	0.0251†
AIC	7440.937	7429.232	3050.121	3029.604	2268.674	2234.844	4719.888	4716.869
BIC	7459.803	7454.387	3068.991	3054.764	2287.544	2260.005	4738.766	4742.040
Harrell’s C-index	0.695	0.7008	0.767	0.7867	0.753	0.7844	0.751	0.7535
IDI	−	0.014 (0.002–0.018)	−	0.010 (0.001–0.020)	−	0.028 (0.004–0.039)	−	0.018 (0.001–0.027)
Continuous NRI	−	0.127 (0.019–0.151)	−	0.132 (0.015–0.169)	−	0.308 (0.035–0.410)	−	0.174 (0.016–0.235)

**2-year**	**MACE**		**All-cause death**		**Cardiovascular death**		**Heart failure**	
Model	Reference*	Reference + TMC	Reference*	Reference + TMC	Reference*	Reference + TMC	Reference*	Reference + TMC
Likelihood ratio test	−	<0.0001†	−	<0.0001†	−	<0.0001†	−	0.0023†
AIC	9538.074	9515.52	4486.232	4461.356	2978.614	2943.09	5318.205	5310.88
BIC	9556.940	9540.675	4505.102	4486.516	2997.484	2968.251	5337.083	5336.050
Harrell’s C-index	0.6870	0.6957	0.7557	0.7716	0.7517	0.7765	0.7582	0.7611
IDI	−	0.019 (0.003–0.023)	−	0.012 (0.002–0.017)	−	0.021 (0.002–0.038)	−	0.024 (0.005–0.036)
Continuous NRI	−	0.134 (0.028–0.163)	−	0.130 (0.021–0.159)	−	0.178 (0.016–0.245)	−	0.185 (0.030–0.249)

**3-year**	**MACE**		**All-cause death**		**Cardiovascular death**		**Heart failure**	
Model	Reference*	Reference + TMC	Reference*	Reference + TMC	Reference*	Reference + TMC	Reference*	Reference + TMC
Likelihood ratio test	−	<0.0001†	−	<0.0001†	−	<0.0001†	−	0.0038†
AIC	11558.130	11526.78	6107.703	6073.171	3610.376	3569.662	6294.361	6287.984
BIC	11576.990	11551.930	6126.574	6098.331	3629.246	3594.822	6313.238	6313.154
Harrell’s C-index	0.6799	0.6907	0.7405	0.7573	0.7447	0.7707	0.7599	0.7626
IDI	−	0.022 (0.004–0.029)	−	0.013 (0.001–0.017)	−	0.020 (0.001–0.020)	−	0.024 (0.002–0.031)
Continuous NRI	−	0.138 (0.026–0.177)	−	0.128 (0.012–0.156)	−	0.166 (0.032–0.237)	−	0.186 (0.019–0.216)

The values in parentheses are 95% confidence intervals. *Reference model includes the Grace score, TnI and NT-proBNP. †Compared with the reference model.

AIC = Akaike information criterion; BIC = Bayesian information criterion; IDI = Integrated discrimination improvement; NRI = Net reclassification improvement; TMC = Trp metabolites combination.

## Discussion

### Primary findings

We reported that the TMC score was associated with increased in the risk of MACE, all-cause death, cardiovascular death, and HF in the cohort with 4071 patients with AMI. Among subsets with 1044 plasma samples collected during the follow-up visit, the Trp metabolites in most participants were stable during the acute and chronic phases after AMI and the associations between second TMC scores and adverse events were repeatable. In the stratification analysis of the binary TMC scores at both points, the First_high_Second_high_ group had the highest risk of poor outcomes. TMC score was added to the Grace 2.0 model, which improved the classification of the risk of outcomes and validated prognostic biomarkers of model performance (Graphical abstract).

### Comparison with previous studies

Previous evidence demonstrated that Kyn was a superior marker for children heart dysfunction and preclinical HF, compared to traditional indicators NT-proBNP and TnI [10], [23]. However, a US study found no associations between coronary heart disease and plasma IPA or Kyn metabolites, which might be explained by the immunoinflammatory response [11]. Considering that antibiotics are major disruptors of gut microbiota, participants with recent antibiotic and probiotic usage were excluded in our study. Supplementation of certain microbes recovered the gut microbiota, reduced plasma Kyn and alleviated ventricular remodelling [24]. Also, altering the gut microbiome composition and supplementing the diet with IPA, can protect against diastolic dysfunction in HF [16]. ILA, as another dietary Trp and gut microbe-generated metabolite, remained significantly associated with MACE [18]. These are consistent with our results and conclusions. 5-HT was shown to have a deleterious role in AMI and other inflammatory diseases [25]. We also measured plasma melatonin level, which was validated cardioprotection against ischemia/reperfusion, cardiomyopathies, atherosclerosis and cardiotoxicity [26]. But no association between melatonin and MACE was found (Supplemental Fig. 1). Patients with AMI had lower circulating melatonin levels at 21.9 pg/mL [27]. In our result, less than half of the total samples were detected using LC-MS/MS. IAA and serotonin were not associated with MACE, so they were not considered in the TMC equation either. High-fat diet leaded to intestinal inflammation and barrier injury, then shifted Trp metabolism from microbiota-derived indole metabolites towards Kyn and serotonin pathways [25]. These supported our idea that a single indicator was not enough to elucidate the associations between abnormal Trp catabolism and the prognosis of AMI.

### Robustness of the TMC score

Previous studies have reported that combining multiple biomarkers has better performance than using a single biomarker [28]. MVX multimarker scores aggregating six biomarkers (GlycA, small HDL, valine, leucine, isoleucine and citrate), can effectively predict cardiovascular mortality and HF events [29], [30]. We believe that our study broadens the knowledge on the relationships between disturbed Trp catabolism and adverse outcomes. Considering the high mortality, cardiovascular mortality, and HF events after AMI within 30 days, we repeated the Cox regression analyses for patients who survived more than 30 days, and TMC scores were still significantly associated with outcomes (Supplemental Table 3). We observed that higher TMC scores were associated with worse EF, then replaced NT-proBNP with EF in Cox regression models, but all associations persisted, even with incident HF (Supplemental Table 4). Blood samples collected from the outpatient follow-up were quantified and scored. Compared to those collected from AMI onset, Kyn and ILA levels decreased but still had associations with outcomes (Supplemental Fig. 3 and Table 3). These results illustrated the robustness of the TMC score in assessing the prognosis of AMI.

### Clinical interpretation

In the second plasma test, the levels of Kyn and ILA as CVD risk factors decreased slightly (Supplemental Fig. 3), which may be because the patients were not in the acute phase of AMI. We compared two TMC regression coefficients and found that the proportion of Kyn in the formula increased (Supplemental Fig. 7). This is consistent with the fact that the majority of free Trp is degraded through the Kyn pathway and generates a range of inflammation and immune response metabolites [31]. More than 95 % of Trp is metabolized along the Kyn pathway to yield nicotinamide and NAD^+^[32]. During the course of many diseases, the intracellular levels of Trp and NAD^+^ can fall if the Kyn pathway is stressed by inflammation or metabolic imbalance. There have been a few studies of NAD^+^ boosters in treating CVD or metabolic diseases [33], [34]. The Kyn-AhR axis has been one of the links between chronic inflammation and tumor progression, which may also become a potential target for AMI therapy [35]. Aloe Emodin, as a traditional Chinese medicine, alleviates radiation-induced heart disease via mitigating Kyn metabolic disruption [36]. A clinical trial showed that the association between plasma Kyn metabolites and the risk of HF was modified by the Mediterranean diet [6]. Indoleamine 2,3-dioxygenase-1 (IDO-1), a rate-limiting enzyme of kynurenine biogenesis, was proved to be a novel therapeutic target for post-vascular injury thrombosis in chronic kidney disease (CKD) [37]. Meanwhile, plasma ILA can potentially be used as biomarkers of CKD and inflammatory status [38], which means the TMC score might be an effective predictor of kidney injury but need further investigation. SGLT2i improved clinical outcomes in HFrEF and influenced Trp metabolism, including 32 % lower Kyn and lower pro-inflammatory cytokines [39]. In both HFpEF mouse models and patients, IPA protected against diastolic dysfunction, metabolic remodeling, oxidative stress and inflammation [6], [16]. Administration of irbesartan reduced intestinal inflammation, and restored Trp metabolism in irritable bowel syndrome mice [40]. More evidence has shown that anti-inflammatory therapy and anti-HF medication improve Trp metabolism and CVD health. Kyn and ILA may be potential targets for the treatment of thrombosis and CKD. Based on the 1-year mortality risk model (Grace Version 2.0) [22], we added cardiac biomarkers including TnI and NT-proBNP, and TMC score to predict outcomes in AMI patients 1-, 2-, and 3-year after discharge. In terms of MACE risk, all-cause mortality and cardiovascular mortality, the addition of TMC showed significant improvement in predictive efficacy (Table 4). These provided evidence on the relationship between Trp and prognosis, and certain diatery and drug intervention may improve adverse outcomes.

### Strengths and limitations

Our study had several strengths. First, we developed a multi-marker score to investigate Trp catabolism dysfunction in an AMI cohort, which was built within a decade. Our cohort were adapted to current epidemiologic characteristics, management and stratification guidelines. Second, we adopted a second measurement of plasma samples collected from outpatient follow-up and performed several sensitivity analyses. Robustness of TMC score and its prognostic value were validated in this AMI cohort. Our study also had several limitations. First, we could not exclude residual confounding, as in other observational studies. Our cohort was predominantly of Asian ancestry and from Northeast China, which limited the generalizability of our findings in other regions and populations. Second, Trp catabolism was influenced by many exogenous factors, including medications, diet, and infections, which might reduce its accuracy as a predictor. We did not profile the full spectrum of Trp metabolic pathways. The metabolites of the 5-HT pathway were at very low levels in the blood and not associated with adverse outcomes. This may attribute to only a small number of plasma samples detected by LC-MS/MS technology. Radioimmunoassays and ELISA have predominated in measuring melatonin [41]. However, our previous research results suggest a protective effect of melatonin in septic cardiomyopathy [7].

## Conclusion

We found that four Trp pathway metabolites were associated with adverse outcomes after AMI separately and additively, supporting the vital role of Trp catabolism in the progression of post-AMI and the importance of risk management for AMI patients. The TMC score we established based on plasma tests combined with the Grace score, adds incremental value to AMI prognosis, providing a simple tool for early risk assessment of AMI patients.

## Funding support

This work was supported by the National Natural Science Foundation of China (62135002, 82170262 and 82200396), National Key R&D Program (No. 2016YFC1301100), and the Fund of Key Laboratory of Myocardial Ischemia, Ministry of Education (KF202217).

## CRediT authorship contribution statement

**Ye Wang:** Writing – original draft, Methodology. **Pengyan Wu:** Methodology, Software. **Zhanchao Chen:** Data curation, Validation. **Zhaoying Li:** Data curation, Validation. **Yini Wang:** Data curation, Validation. **Miao Yan:** Supervision. **Yiying Zhang:** Supervision. **Shanjie Wang:** Writing – review & editing. **Shaohong Fang:** Conceptualization. **Bo Yu:** Conceptualization.

## Data availability

The data described in the manuscript, code book, and analytic code will be made available upon request pending.

## Declaration of competing interest

The authors declare that they have no known competing financial interests or personal relationships that could have appeared to influence the work reported in this paper.
