# Supplementary material for: Prognostic value of tryptophan catabolism-base scores in acute myocardial infarction patients
Source: J Adv Res. 2025 Mar 25;79:563–70. doi: 10.1016/j.jare.2025.03.025 (PMC12766179; doi:10.1016/j.jare.2025.03.025)

**Supplemental Materials**

## Supplemental Method

### Targeted mass spectrometry analyses

For the metabolomics analysis, fasting blood samples were collected in EDTA-containing tubes and stored in freezers at -80 °C. Stable-isotope-dilution LC-MS/MS was developed to specifically quantify metabolites in human plasma. Briefly, ice-cold methanol (250μL) containing internal standard (L-tryptophan-d5) was added to plasma samples (50μL), followed by vortexing and centrifugation. The supernatant was lyophilized in a centrifugal vacuum evaporator at 0 °C. The sample was reconstituted in 25μL of 50% (vol/vol) H_2_O/methanol, followed by vortexing and centrifugation. The clear supernatant was transferred to glass vials with micro inserts, and LC-MS/MS analysis was based on an UltiMate 3000 UHPLC system (Thermo Fisher) coupled to a TSQ Quantis triple quadruple mass spectrometer (Thermo Fisher). For chromatographic separation, an ACQUITY HSS T3 column (i.d., 2.1×100 mm, 1.8μm) (Waters) was used. The temperatures of the column and autosampler were set at 30 °C and 4 °C, respectively. The sample injection volume was 1 µL. The mobile phase consisted of 0.1% (vol/vol) formic acid/H_2_O (A) and 0.1% (vol/vol) formic acid/acetonitrile (B). The flow rate was set at 0.3 mL/min. A 8-min elution gradient was performed in Table 1. Mass spectrometric detection was carried out in positive and negative electrospray ionization mode, employing selected reaction monitoring (SRM) to target speciﬁc m/z transitions for each analyte, thereby ensuring selectivity and minimizing matrix interferences. Detailed mass spectrometry conditions for analytes were shown in Table 2. Instrumental parameters, such as declustering potential and collision energy, were ﬁnely tuned to achieve the highest sensitivity and speciﬁcity for analyte detection. Data acquisition, management, and analysis were conducted using the integrated Thermo Scientiﬁc software, providing a robust platform for peak integration, quantitative analysis, and ensuring data integrity in compliance with established analytical standards.

### Method validation

The bioanalytical method employed in this study underwent validation in accordance with compliance requirements described in the FDA guidance document titled “M10 Bioanalytical Method Validation and Study Sample Analysis”. The validation criteria consisted of parameters including lower limit of quantification (LLOQ), linearity, accuracy, precision, recovery, matrix effect and stability. The acceptability criteria of linearity were that the correlation coefficient should be >0.99 and the standard concentration at each concentration point should deviate from the nominal value within ± 15%, except for a maximum acceptable deviation of ± 20% for LLOQ (Table 2). LLOQ was calculated with a signal-to-noise ratio (S/N) of at least 3 and 10, respectively. Intra-day and inter-day accuracy and precision were assessed by analysing low, medium, and high levels of QC samples on three days, with each level performed five times on the same day. In terms of accuracy, the acceptance criteria for QC samples were within 85–115%. In terms of precision, measured as relative standard deviation (RSD), the acceptance criteria for QC samples were <15 % (Table 3).

The recovery experiment and matrix effect were performed at three (low, medium, and high) QC levels, with each level being performed five times. Recovery was calculated by comparing the analytical results of the extracted samples with those of the blank samples spiked with the analytes post extraction. The matrix effect was calculated by comparing the peak areas of each analyte added to the extracted biological matrix with methanol containing equivalent analytes. The acceptance criteria for recovery and matrix effect were in the range of 85–115% and RSD ≤ 15% (Table 4).

The stability of each analyte was also assessed at the low, medium, and high QC levels, including bench-top stability (10 hours at room temperature), autosampler stability (12 hours at 4°C), freeze-thaw stability (three cycles), and long-term stability (30 days at −80°C). QC samples at each condition were compared to freshly prepared samples and were considered stable if the accuracy was within 85–115% and RSD ≤ 15% (Table 5).

### Table 1 Reversed-phase UHPLC-MS/MS gradient

| **Time (min)** | **Flow rate (ml/min)** | **Mobile phase A (%)** | **Mobile phase B (%)** |
| --- | --- | --- | --- |
| Initial | 0.3 | 90 | 10 |
| 1.0 | 0.3 | 90 | 10 |
| 5.0 | 0.3 | 40 | 60 |
| 5.5 | 0.3 | 5 | 95 |
| 6.0 | 0.3 | 5 | 95 |
| 6.2 | 0.3 | 90 | 10 |
| 8.0 | 0.3 | 90 | 10 |

### Table 2 Mass spectrometric conditions for analyte quantitation

| **Analyte** | **t_R_**  **(min)** | **Calibration Curve** | **R^2^** | **Linear range(ng/mL)** | **LLOQ**  **(ng/mL)** | **SRM-1** | | **SRM-2** | |
| --- | --- | --- | --- | --- | --- | --- | --- | --- | --- |
|  |  |  |  |  |  | **Transition** | **CE(V)** | **Transition** | CE(V) |
| Indolylpropionic acid | 5.31 | Y=889.3X-309.1 | 0.9993 | 1.953-1000 | 1.953 | 190.138>130.042 | +15.49 | 190.138>149.042 | +6.56 |
| Indole-3-lactic acid | 4.42 | Y=231X-1537 | 0.9993 | 1.953-1000 | 1.953 | 204.062>158.054 | -14.6 | 204.062>186.113 | -12.96 |
| L-tryptophan | 2.69 | Y=0.002589X+0.06784 | 0.9931 | 1.953-1000 | 1.953 | 205>146.042 | +17.43 | 205>188.125 | +9.89 |
| L-kynurenine | 1.61 | Y=1359X+2880 | 0.9994 | 1.953-1000 | 1.953 | 209.1>146.042 | +18.61 | 209.1>192.125 | +8.83 |

### Table 3 Intra-day and inter-day accuracy and precision

| **Analyte** | **Concentration type** | **Intra-Day(n=5)** | | **Inter-Day(n=15)** | |
| --- | --- | --- | --- | --- | --- |
|  |  | **Accuracy (%)** | **Precision (RSD%)** | **Accuracy (%)** | **Precision (RSD%)** |
| IPA | Low QC | 107.04% | 7.22% | 99.59% | 9.27% |
|  | Medium QC | 92.57% | 8.34% | 95.07% | 3.82% |
|  | High QC | 92.67% | 3.14% | 95.67% | 2.71% |
| ILA | Low QC | 96.97% | 7.61% | 93.38% | 3.34% |
|  | Medium QC | 103.14% | 5.54% | 101.99% | 2.85% |
|  | High QC | 97.39% | 3.95% | 99.96% | 2.46% |
| Tryptophan | Low QC | 90.89% | 13.04% | 96.30% | 5.90% |
|  | Medium QC | 93.84% | 6.48% | 97.27% | 3.50% |
|  | High QC | 107.27% | 4.84% | 104.58% | 2.94% |
| Kynurenine | Low QC | 109.93% | 8.59% | 107.91% | 3.19% |
|  | Medium QC | 98.12% | 4.15% | 99.93% | 3.87% |
|  | High QC | 99.36% | 5.13% | 101.53% | 3.35% |

### Table 4 Recovery experiment and matrix effect

| **Analyte** | **Concentration type** | **Recovery(n=15）** | | **Matrix effect(n=15）** | |
| --- | --- | --- | --- | --- | --- |
|  |  | **Mean (%)** | **RSD (%)** | **Mean (%)** | **RSD (%)** |
| IPA | Low QC | 91.61% | 3.24% | 111.66% | 10.40% |
|  | Medium QC | 97.06% | 4.04% | 101.14% | 6.76% |
|  | High QC | 89.37% | 4.14% | 95.97% | 5.05% |
| ILA | Low QC | 93.81% | 5.22% | 103.29% | 10.83% |
|  | Medium QC | 88.51% | 11.73% | 95.37% | 5.31% |
|  | High QC | 94.70% | 4.66% | 89.97% | 3.73% |
| Tryptophan | Low QC | 94.40% | 7.68% | 99.66% | 11.92% |
|  | Medium QC | 92.89% | 7.79% | 110.80% | 7.91% |
|  | High QC | 95.09% | 3.00% | 86.33% | 1.72% |
| Kynurenine | Low QC | 95.50% | 5.20% | 101.70% | 8.52% |
|  | Medium QC | 96.22% | 5.49% | 99.69% | 6.51% |
|  | High QC | 99.07% | 1.40% | 88.29% | 3.36% |

### Table 5 Short-term stability, long-term stability, autosampler stability and freeze-thaw stability

| **Analyte** | **Concentration type** | **Short-term Stability** | | **Long-term Stability** | | **Autosampler Stability** | | **Freeze-thaw stability** | |
| --- | --- | --- | --- | --- | --- | --- | --- | --- | --- |
|  |  | **Mean(%)** | **RSD(%)** | **Mean(%)** | **RSD(%)** | **Mean(%)** | **RSD(%)** | **Mean(%)** | **RSD(%)** |
| IPA | Low QC | 104.07% | 7.39% | 102.87% | 9.09% | 96.98% | 3.74% | 98.93% | 7.34% |
|  | Medium QC | 100.61% | 3.64% | 90.35% | 4.08% | 96.32% | 4.96% | 92.82% | 2.65% |
|  | High QC | 99.82% | 3.09% | 91.54% | 6.50% | 89.13% | 3.87% | 90.83% | 2.56% |
| ILA | Low QC | 93.47% | 11.76% | 96.92% | 7.38% | 99.94% | 8.99% | 99.87% | 9.86% |
|  | Medium QC | 97.09% | 5.28% | 91.02% | 5.37% | 103.57% | 0.85% | 94.79% | 4.85% |
|  | High QC | 94.75% | 4.54% | 97.78% | 3.82% | 95.02% | 5.25% | 87.73% | 2.09% |
| Tryptophan | Low QC | 99.06% | 7.43% | 87.98% | 2.50% | 97.52% | 1.43% | 98.14% | 2.59% |
|  | Medium QC | 93.22% | 6.58% | 101.80% | 5.04% | 97.72% | 2.88% | 88.45% | 4.07% |
|  | High QC | 107.05% | 4.25% | 88.34% | 2.01% | 90.75% | 2.24% | 88.95% | 3.35% |
| Kynurenine | Low QC | 102.62% | 8.73% | 95.91% | 8.09% | 90.56% | 1.28% | 95.19% | 5.42% |
|  | Medium QC | 98.57% | 2.74% | 87.92% | 1.78% | 96.43% | 3.67% | 93.13% | 3.58% |
|  | High QC | 105.92% | 2.83% | 88.86% | 4.53% | 93.25% | 7.41% | 86.08% | 3.25% |

## Supplemental Table 1 Spearman correlations in metabolites

|  | **Trp** | **Kyn** | **IPA** | **ILA** |
| --- | --- | --- | --- | --- |
| **Trp** | 1.0000 |  |  |  |
| **Kyn** | 0.3838 | 1.0000 |  |  |
| **IPA** | 0.1592 | 0.1707 | 1.0000 |  |
| **ILA** | 0.3604 | 0.5258 | 0.1815 | 1.0000 |

Correlation coefficients ≥ 0.1592 are significant (p<0·001). Trp = Tryptophan; Kyn = Kynurenine; IPA = indole-3-propionic acid; ILA = indole-3-lactic acid.

## Supplemental Table 2 Association between the second TMC score tertiles and incident MACE

|  | **Tertile 1 (n = 348)** | **Tertile 2 (n = 348)** | **Tertile 3 (n = 348)** | **P trend** | **Increase per SD** | **P Value** |
| --- | --- | --- | --- | --- | --- | --- |
| **MACE**  **(n = 245)** |  | | | | | |
| Unadjusted | 1 | 1.21 (0.87-1.69) | 1.83 (1.34-2.50) | <0.001 | 1.36 (1.20-1.53) | <0.001 |
| Model 1 | 1 | 1.21 (0.87-1.70) | 1.81 (1.32-2.47) | <0.001 | 1.33 (1.18-1.50) | <0.001 |
| Model 2 | 1 | 1.14 (0.81-1.61) | 1.50 (1.08-2.10) | 0.014 | 1.21 (1.06-1.38) | 0.006 |

The values are hazard ratios (95% confidence intervals). Model 1: Adjusted for age and sex. Model 2: Adjusted for variables in Model 1 plus hypertension, diabetes mellitus, smoking status, BMI, previous myocardial infarction, previous stroke, PCI, TnI, NTpro-BNP, hs-CRP and CKD-EPI.

## Supplemental Table 3 Association between TMC tertiles and outcomes among patients survived more than 30 days

|  | **Tertile 1 (n = 1357)** | **Tertile 2 (n = 1357)** | **Tertile 3 (n = 1357)** | **Ptrend** | **Increase per SD** | **P Value** |
| --- | --- | --- | --- | --- | --- | --- |
| **MACE (n = 922)** |  | | | | | |
| Unadjusted | 1 | 1.37 (1.15-1.64) | 2.02 (1.71-2.39) | <0.001 | 1.38 (1.29-1.46) | <0.001 |
| Model 1 | 1 | 1.34 (1.12-1.59) | 1.87 (1.58-2.21) | <0.001 | 1.33 (1.25-1.42) | <0.001 |
| Model 2 | 1 | 1.29 (1.08-1.53) | 1.66 (1.40-1.97) | <0.001 | 1.25 (1.17-1.34) | <0.001 |
| **Death (n = 514)** |  | | | | | |
| Unadjusted | 1 | 1.22 (0.96-1.55) | 2.38 (1.92-2.96) | <0.001 | 1.55 (1.43-1.67) | <0.001 |
| Model 1 | 1 | 1.16 (0.91-1.47) | 2.07 (1.66-2.57) | <0.001 | 1.47 (1.36-1.59) | <0.001 |
| Model 2 | 1 | 1.11 (0.87-1.42) | 1.85 (1.48-2.31) | <0.001 | 1.37 (1.26-1.50) | <0.001 |
| **Cardiovascular death (n = 230)** |  | | | | | |
| Unadjusted | 1 | 1.31 (0.89-1.91) | 3.01 (2.15-4.22) | <0.001 | 1.68 (1.51-1.88) | <0.001 |
| Model 1 | 1 | 1.25 (0.86-1.84) | 2.68 (1.91-3.76) | <0.001 | 1.62 (1.45-1.82) | <0.001 |
| Model 2 | 1 | 1.17 (0.80-1.712) | 2.19 (1.55-3.11) | <0.001 | 1.46 (1.29-1.66) | <0.001 |
| **HF (n = 372)** |  | | | | | |
| Unadjusted | 1 | 1.68 (1.27-2.22) | 2.44 (1.86-3.19) | <0.001 | 1.46 (1.33-1.60) | <0.001 |
| Model 1 | 1 | 1.63 (1.23-2.16) | 2.24 (1.71-2.93) | <0.001 | 1.42 (1.29-1.56) | <0.001 |
| Model 2 | 1 | 1.52 (1.15-2.02) | 1.88 (1.43-2.49) | <0.001 | 1.29 (1.16-1.43) | <0.001 |

The values are hazard ratios (95% confidence intervals). Model 1: Adjusted for age and sex. Model 2: Adjusted for variables in Model 1 plus hypertension, diabetes mellitus, smoking status, BMI, previous myocardial infarction, previous stroke, PCI, TnI, NTpro-BNP, hs-CRP and CKD-EPI. TMC = Trp metabolites combination.

## Supplemental Table 4 Association between TMC tertiles and outcomes with EF replacing NT-proBNP

|  | **Tertile 1 (n = 1357)** | **Tertile 2 (n = 1357)** | **Tertile 3 (n = 1357)** | **Ptrend** | **Increase per SD** | **P Value** |
| --- | --- | --- | --- | --- | --- | --- |
| **MACE (n = 1212)** |  | | | | | |
| Unadjusted | 1 | 1.37 (1.17-1.60) | 2.06 (1.77-2.38) | <0.001 | 1.38 (1.31-1.46) | <0.001 |
| Model 1 | 1 | 1.33 (1.14-1.55) | 1.88 (1.62-2.18) | <0.001 | 1.33 (1.26-1.41) | <0.001 |
| Model 2 | 1 | 1.18 (1.00-1.38) | 1.51 (1.29-1.75) | <0.001 | 1.21 (1.15-1.29 | <0.001 |
| **Death (n = 666)** |  | | | | | |
| Unadjusted | 1 | 1.25 (0.99-1.56) | 2.51 (2.05-3.07) | <0.001 | 1.59 (1.48-1.71) | <0.001 |
| Model 1 | 1 | 1.18 (0.94-1.48) | 2.17 (1.77-2.66) | <0.001 | 1.51 (1.40-1.63) | <0.001 |
| Model 2 | 1 | 1.10 (0.88-1.38) | 1.85 (1.50-2.29) | <0.001 | 1.41 (1.31-1.53) | <0.001 |
| **Cardiovascular death (n = 365)** |  | | | | | |
| Unadjusted | 1 | 1.31 (0.93-1.84) | 3.28 (2.44-4.40) | <0.001 | 1.77 (1.61-1.94) | <0.001 |
| Model 1 | 1 | 1.25(0.89-1.76) | 2.88 (2.14-3.87) | <0.001 | 1.71 (1.55-1.88) | <0.001 |
| Model 2 | 1 | 1.11 (0.79-1.56) | 2.26 (1.66-3.06) | <0.001 | 1.56 (1.40-1.74) | <0.001 |
| **HF (n = 559)** |  | | | | | |
| Unadjusted | 1 | 1.62 (1.28-2.05) | 2.37 (1.89-2.97) | <0.001 | 1.44 (1.33-1.56) | <0.001 |
| Model 1 | 1 | 1.57 (1.24-1.99) | 2.16 (1.72-2.71) | <0.001 | 1.39 (1.28-1.51) | <0.001 |
| Model 2 | 1 | 1.24 (0.97-1.57) | 1.46 (1.15-1.85) | 0.001 | 1.17 (1.07-1.28) | <0.001 |

The values are hazard ratios (95% confidence intervals). Model 1: Adjusted for age and sex. Model 2: Adjusted for variables in Model 1 plus hypertension, diabetes mellitus, smoking status, BMI, previous myocardial infarction, previous stroke, PCI, TnI, EF, hs-CRP and CKD-EPI. TMC = Trp metabolites combination.

## Supplemental Table 5 Association between TMC tertiles and outcomes treating death as a competing risk

|  | **Tertile 1 (n = 1357)** | **Tertile 2 (n = 1357)** | **Tertile 3 (n = 1357)** | **Ptrend** |
| --- | --- | --- | --- | --- |
| **Cardiovascular death (n = 365)** |  | | | |
| Unadjusted | 1 | 1.30 (0.93-1.82) | 3.20 (2.38-4.30) | <0.001 |
| Model 1 | 1 | 1.25 (0.89-1.75) | 2.80 (2.08-3.77) | <0.001 |
| Model 2 | 1 | 1.16 (0.82-1.64) | 2.27 (1.67-3.07) | <0.001 |
| **HF (n = 559)** |  | | | |
| Unadjusted | 1 | 1.61 (1.27-2.03) | 2.29 (1.82-2.86) | <0.001 |
| Model 1 | 1 | 1.56 (1.23-1.97) | 2.08 (1.66-2.61) | <0.001 |
| Model 2 | 1 | 1.42 (1.12-1.81) | 1.67 (1.32-2.11) | <0.001 |

The values are hazard ratios (95% confidence intervals). Model 1: Adjusted for age and sex. Model 2: Adjusted for variables in Model 1 plus hypertension, diabetes mellitus, smoking status, BMI, previous myocardial infarction, previous stroke, PCI, TnI, NTpro-BNP, hs-CRP and CKD-EPI. TMC = Trp metabolites combination.

## Supplemental Table 6 Association between TMC tertiles and predicted (actual) event rate at various timing

| **Outcomes** | **Group** | **6 months (%)** | **1 year (%)** | **2 years (%)** | **3 years (%)** |
| --- | --- | --- | --- | --- | --- |
| **MACE** | Tertile 1 | 7.50 (7.66) | 9.25 (9.14) | 11.68 (9.14) | 14.11 (13.56) |
|  | Tertile 2 | 9.98 (9.87) | 12.24 (12.16) | 15.36 (12.16) | 18.43 (18.50) |
|  | Tertile 3 | 15.38 (15.18) | 18.60 (18.64) | 22.93 (18.64) | 27.06 (26.97) |
| **All-cause death** | Tertile 1 | 2.89 (3.02) | 3.71 (3.61) | 5.16 (3.61) | 6.82 (7.00) |
|  | Tertile 2 | 4.23 (3.61) | 5.41 (4.72) | 7.47 (4.72) | 9.81 (9.21) |
|  | Tertile 3 | 8.15 (8.62) | 10.26 (10.98) | 13.81 (10.98) | 17.66 (17.39) |
| **Cardiovascular death** | Tertile 1 | 2.03 (2.21) | 2.46 (2.51) | 3.09 (2.51) | 3.67 (3.83) |
|  | Tertile 2 | 3.30 (2.43) | 4.00 (2.95) | 5.00 (2.95) | 5.92 (4.79) |
|  | Tertile 3 | 7.35 (7.96) | 8.78 (9.58) | 10.80 (9.58) | 12.58 (12.75) |
| **Heart failure** | Tertile 1 | 4.50 (4.35) | 5.39 (5.01) | 6.15 (5.01) | 7.37 (6.63) |
|  | Tertile 2 | 6.44 (6.34) | 7.69 (7.59) | 8.74 (7.59) | 10.43 (10.24) |
|  | Tertile 3 | 10.99 (10.46) | 12.97 (12.31) | 14.61 (12.31) | 17.19 (15.77) |

## Supplemental Table 7 The second TMC score components and regression coefficients

|  | **HR** | **p Value** | **Coefficient** |
| --- | --- | --- | --- |
| ILA | 1.15 (1.00-1.33) | 0.050 | 0.143 |
| IPA | 0.88 (0.78-1.00) | 0.046 | -0.128 |
| KYN | 1.44 (1.12-1.84) | 0.004 | 0.364 |
| TRP | 0.92 (0.86-0.99) | 0.027 | -0.081 |

## Supplemental Table 8 Baseline characteristics of AMI patients in high-low stratifications of two TMC scores

|  | Total | **First_low_Second_low_ (n = 401)** | **First_high_Second_low_ (n = 121)** | **First_low_Second_high_  (n = 121)** | **First_high_Second_high_ (n = 401)** | P Value |
| --- | --- | --- | --- | --- | --- | --- |
| **Demographics** |  |  |  |  |  |  |
| Age, years | 57.2 ± 11.3 | 56.5 ± 11.4 | 54.9 ± 11.0 | 57.5 ± 11.9 | 58.4 ± 11.0 | 0.009 |
| Male | 787 (75.38) | 295 (73.57) | 98 (80.99) | 82 (67.77) | 312 (77.81) | 0.347 |
| **Cardiovascular risk factors** |  |  |  |  |  |  |
| BMI, kg/m2 | 25.6 ± 3.8 | 25.4 ± 3.5 | 25.6 ± 3.7 | 25.2 ± 3.4 | 25.8 ± 4.2 | 0.155 |
| Current smoker | 711 (68.10) | 261 (65.09) | 84 (69.42) | 81 (66.94) | 285 (71.07) | 0.087 |
| Diabetes Mellitus | 245 (23.47) | 98 (24.44) | 30 (24.79) | 28 (23.14) | 89 (22.19) | 0.428 |
| Hypertension | 553 (52.97) | 188 (46.88) | 61 (50.41) | 66 (54.55) | 238 (59.35) | <0.001 |
| Total cholesterol, mmol/L | 4.57 (3.93, 5.21) | 4.59 (3.98, 5.25) | 4.57 (4.18, 5.11) | 4.48 (3.91, 4.88) | 4.55 (3.90, 5.27) | 0.155 |
| HDL, mmol/L | 1.22 (1.06, 1.42) | 1.22 (1.04, 1.44) | 1.22 (1.06, 1.40) | 1.22 (1.06, 1.46) | 1.22 (1.08, 1.41) | 0.822 |
| LDL, mmol/L | 2.82 (2.31, 3.33) | 2.82 (2.36, 3.38) | 2.82 (2.46, 3.35) | 2.70 (2.34, 3.09) | 2.75 (2.24, 3.32) | 0.070 |
| hs-CRP, mg/L | 5.07 (2.11, 10.90) | 4.82 (2.02, 10.23) | 4.57 (1.86, 10.57) | 4.83 (1.94, 11.14) | 5.42 (2.32, 11.23) | 0.094 |
| CKD-EPI, mL/min/1.73 m2 | 85.2 (70.8, 100.3) | 91.5 (76.5, 105.1) | 88.2 (72.2, 102.0) | 81.5 (64.8, 98.7) | 79.1 (64.7, 93.5) | <0.001 |
| **Medical history** |  |  |  |  |  |  |
| Myocardial infarction | 79 (7.57) | 26 (6.48) | 8 (6.61) | 7 (5.79) | 38 (9.48) | 0.127 |
| Stroke | 183 (17.53) | 64 (15.96) | 21 (17.36) | 23 (19.01) | 75 (18.70) | 0.287 |
| **MI characteristics** |  |  |  |  |  |  |
| EF, % | 58.0 ± 6.9 | 58.5 ± 6.5 | 58.3 ± 6.2 | 58.4 ± 5.9 | 57.2 ± 7.6 | 0.007 |
| Troponin I, ng/L | 36.2 (10.7, 101.8) | 31.8 (8.6, 94.0) | 37.0 (18.0, 125.8) | 36.2 (13.2, 105.5) | 36.2 (10.7, 99.0) | 0.556 |
| NT-ProBNP, pmol/L | 718 (280, 1726.5) | 581 (216, 1563) | 681 (312, 1384) | 805 (405, 1830) | 852 (328, 2083) | 0.225 |
| **Medical treatment** |  |  |  |  |  |  |
| Asprin | 1034 (99.04) | 397 (99.00) | 120 (99.17) | 121 (100) | 396 (98.75) | 0.812 |
| Clopidogrel or ticagrelor | 1039 (99.52) | 400 (99.75) | 119 (98.35) | 120 (99.17) | 400 (99.75) | 0.867 |
| Statins | 1021 (97.80) | 396 (98.75) | 115 (95.04) | 118 (97.52) | 392 (97.76) | 0.479 |
| β-Blocker | 1013 (97.03) | 392 (97.76) | 118 (97.52) | 114 (94.21) | 389 (97.01) | 0.377 |
| ACEI/ARB | 837 (80.17) | 309 (77.06) | 98 (80.99) | 97 (80.17) | 333 (83.04) | 0.040 |
| CAG | 1035 (99.14) | 398 (99.25) | 119 (98.35) | 121 (100) | 397 (99.00) | 0.900 |
| PCI | 903 (86.49) | 356 (88.78) | 98 (80.99) | 100 (82.64) | 349 (87.03) | 0.520 |
| **Follow-up max years** | 5.9 (5.4, 6.3) | 5.8 (5.4, 6.2) | 6.3 (5.6, 6.9) | 6.5 (5.5, 7.0) | 5.9 (5.3, 6.3) | 0.436 |
| TMC components |  |  |  |  |  |  |
| Tryptophan, μg/mL | 9.6 (7.1, 12.9) | 9.0 (6.5, 11.8) | 7.5 (6.2, 11.1) | 10.5 (7.6, 14.2) | 11.1 (8.1, 14.4) | <0.001 |
| Kynurenine, ng/mL | 329.0 (241.1, 441.0) | 237.6 (190.7, 291.0) | 250.4 (209.1, 301.6) | 392.2 (336.4, 468.8) | 445.2 (372.8, 551.3) | <0.001 |
| IPA, ng/mL | 194.3 (89.2, 557.7) | 197.6 (95.3, 664.2) | 135.8 (60.4, 403.8) | 311.7 (130.4, 1064.7) | 189.0 (83.6, 429.9) | 0.012 |
| ILA, ng/mL | 138.9 (98.2, 203.2) | 104.8 (78.5, 133.6) | 133.4 (94.3, 196.1) | 154.9 (105.6, 216.2) | 195.5 (146.7, 255.8) | <0.001 |
| Coronary angiography features |  |  |  |  |  |  |
| Number of diseased vessels |  |  |  |  |  |  |
| 1 vessel, n (%) | 350 (33.52) | 136 (33.92) | 47 (38.84) | 36 (29.75) | 131 (32.67) | 0.739 |
| 2 vessels, n (%) | 420 (40.23) | 165 (41.15) | 46 (38.02) | 52 (42.98) | 157 (39.15) |  |
| 3 vessels, n (%) | 274 (26.25) | 100 (24.94) | 28 (23.14) | 33 (27.27) | 113 (28.18) |  |
| Culprit vessel, n (%) |  |  |  |  |  |  |
| LAD | 481 (46.07) | 171 (42.64) | 55 (45.45) | 52 (42.98) | 203 (50.62) | 0.080 |
| LCX | 268 (25.67) | 119 (29.68) | 32 (26.45) | 36 (29.75) | 81 (20.20) |  |
| RCA | 295 (28.26) | 111 (27.68) | 34 (28.10) | 33 (27.27) | 117 (29.18) |  |
| Door-to-balloon (minutes) | 67 (48, 87) | 69 (51, 86) | 69 (47, 88) | 62 (43, 87) | 67 (49, 86) | 0.321 |

Values are mean (SD), median (IQR) or n (%). BMI=body mass index; CKD-EPI = chronic kidney disease epidemiology collaboration; IPA = indole-3-propionic acid; ILA = indole-3-lactic acid; Trp = tryptophan; Kyn = kynurenine; LAD = left anterior descending; LCX = left circumflex; RCA = right coronary artery.

## Supplemental Figure 1


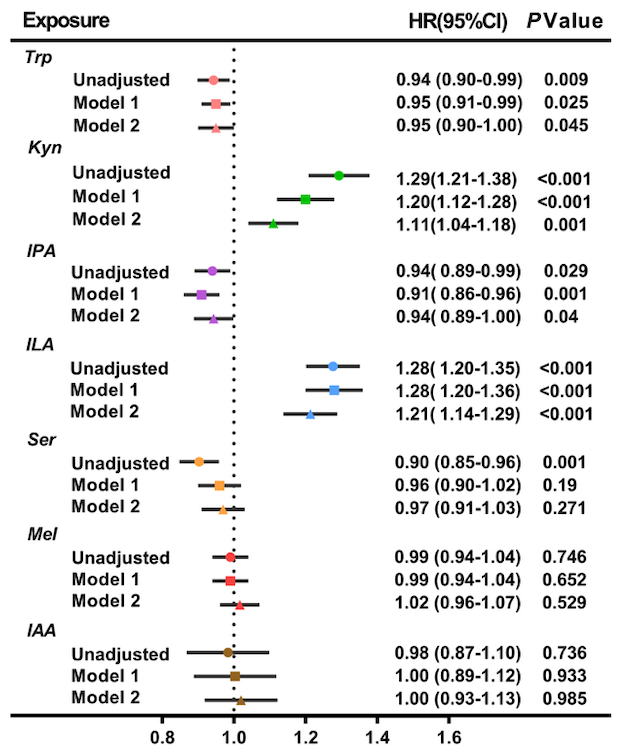


Forest plot for association between Trp metabolites and incident MACE in AMI patients (n=4071). Model 1: Adjusted for age and sex. Model 2: Adjusted for variables in Model 1 plus hypertension, diabetes mellitus, smoking status, BMI, previous myocardial infarction, previous stroke, TnI, NTpro-BNP, hs-CRP and CKD-EPI. Trp = Tryptophan; Kyn = Kynurenine; IPA = indole-3-propionic acid; ILA = indole-3-lactic acid; Ser = serotonin; Mel = melatonin; IAA = Indole acetic acid.

## Supplemental Figure 2


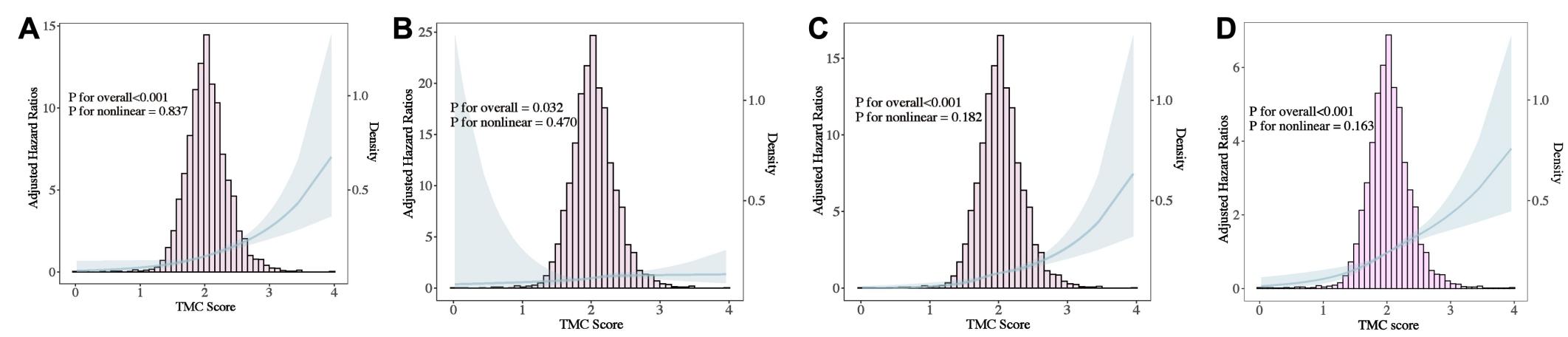


Restricted cubic spline curves and histograms for TMC scores and all-cause mortality (A), cardiovascular mortality (B), HF(C) and MACE(D) in AMI patients (n=4071).

## Supplemental Figure 3


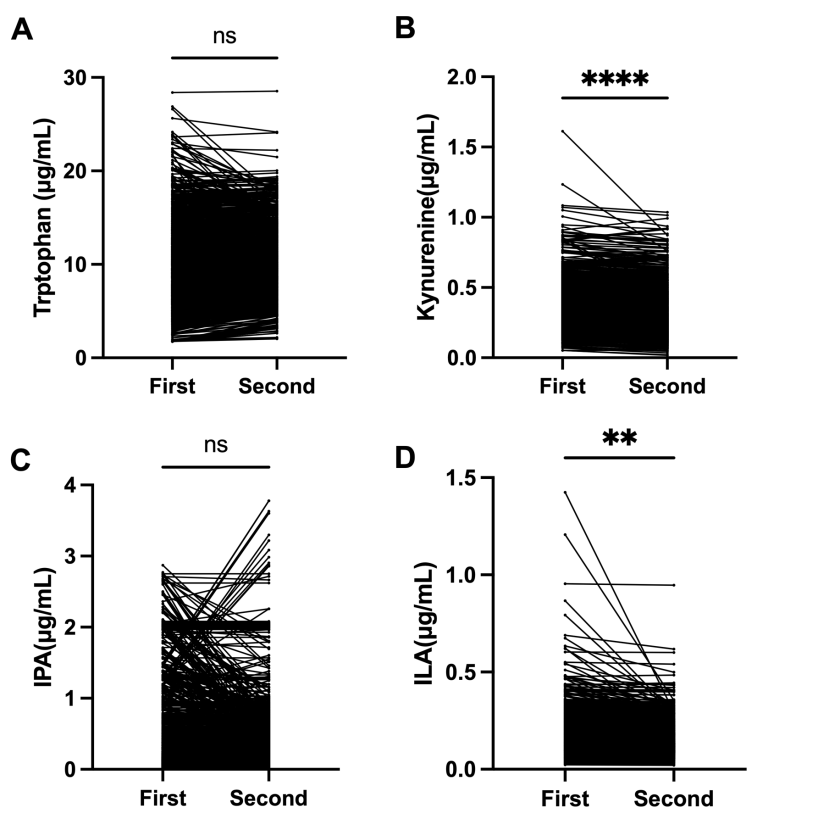


Two measurements of 1044 AMI patients’ plasma tryptophan (A), kynurenine (B), IPA (C) and ILA (D) at baseline and outpatient follow-up.

## Supplemental Figure 4


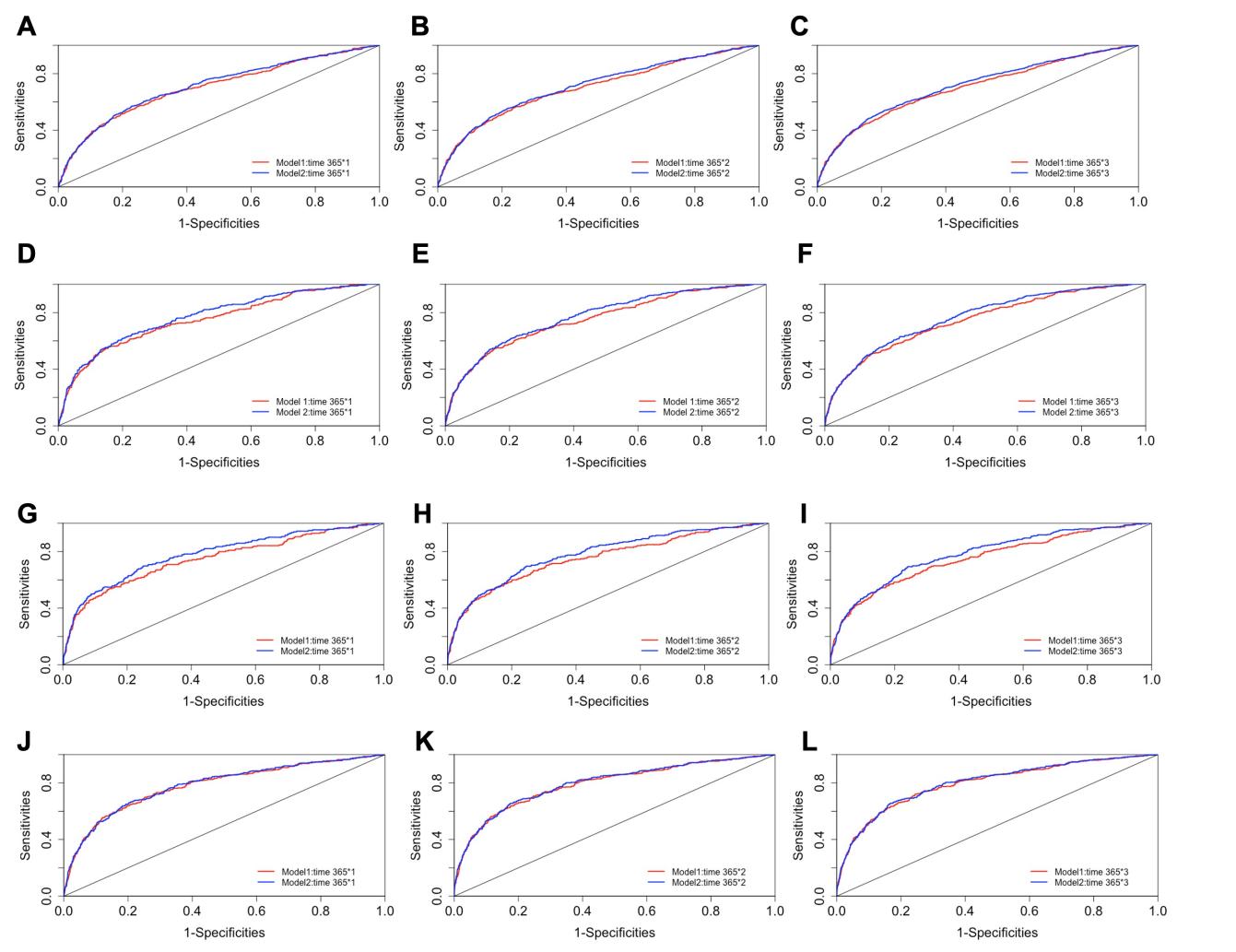


ROC curve analysis of the predictive accuracy of the models for MACE (A-C), all-cause mortality (D-F), cardiovascular mortality (G-I) and HF (J-L) in AMI patients (n=4071).

## Supplemental Figure 5


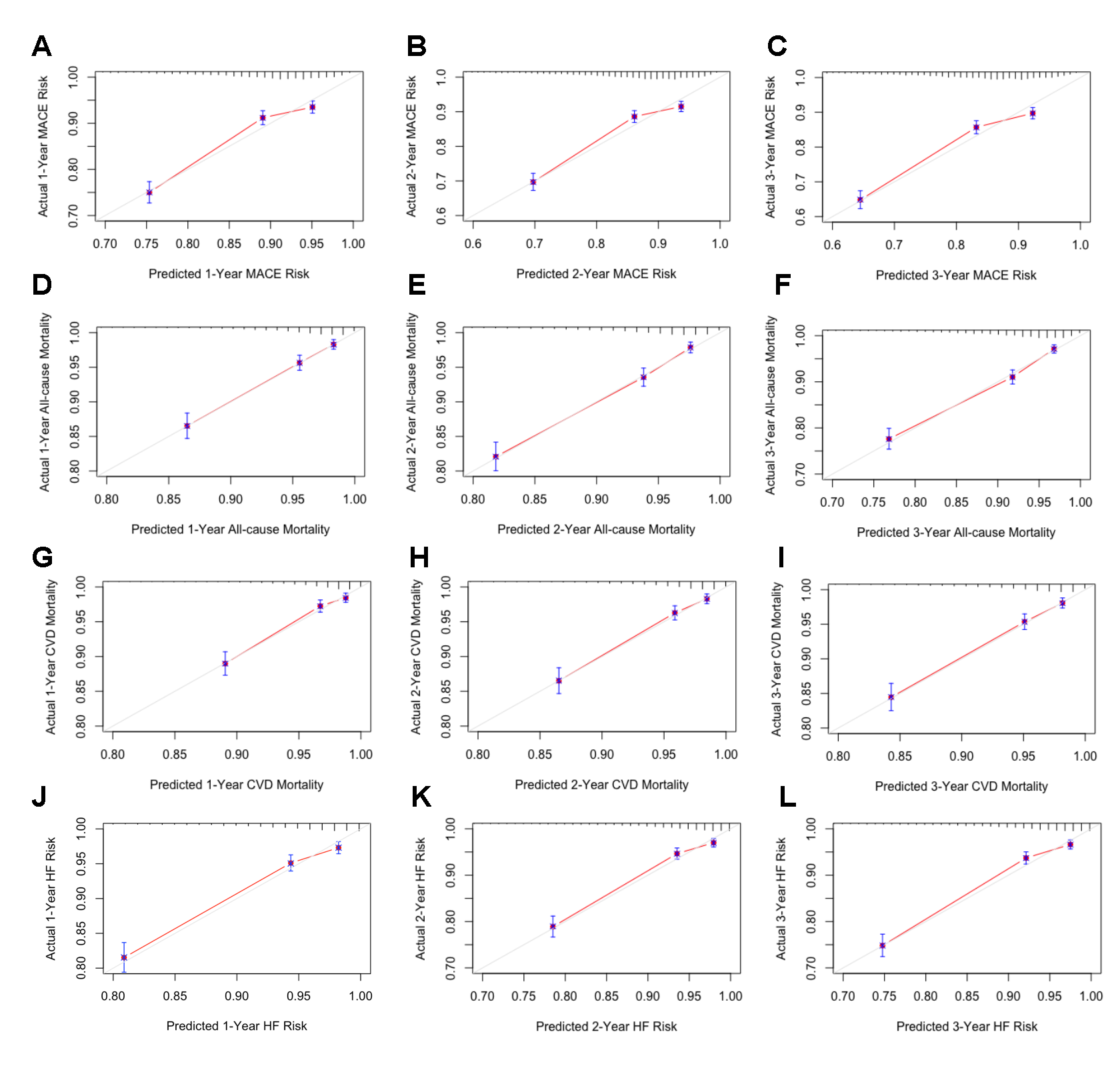


Calibration curves for the predictive accuracy of the models for MACE (A-C), all-cause mortality (D-F), cardiovascular mortality (G-I) and HF (J-L) in AMI patients (n=4071).

## Supplemental Figure 6


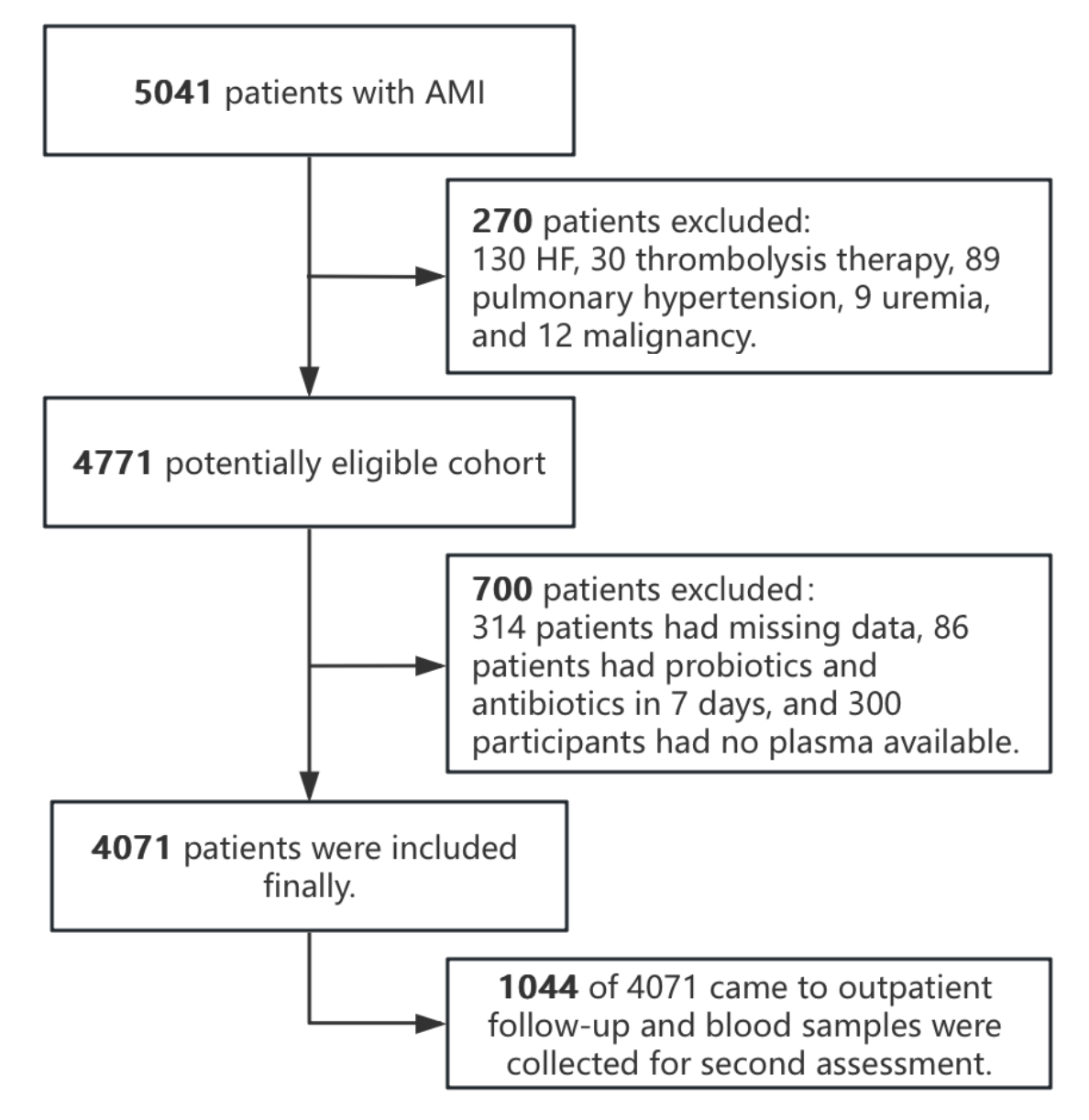


## Supplemental Figure 7


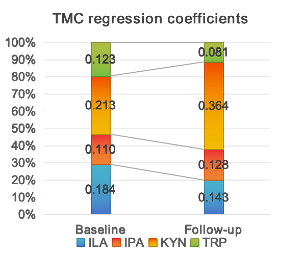

Supplement: Supplementary Data 1 [file mmc1.docx]
